# Modeling the Evolution of Female Meiotic Drive in Maize

**DOI:** 10.1534/g3.117.300073

**Published:** 2017-11-09

**Authors:** David W. Hall, R. Kelly Dawe

**Affiliations:** * Department of Genetics, University of Georgia, Athens, Georgia 30602-7223; † Department of Plant Biology, University of Georgia, Athens, Georgia 30602-7223

**Keywords:** abnormal chromosome 10, autosomal drive, neocentromere, segregation distortion, selfish genetic element

## Abstract

Autosomal drivers violate Mendel’s law of segregation in that they are overrepresented in gametes of heterozygous parents. For drivers to be polymorphic within populations rather than fixing, their transmission advantage must be offset by deleterious effects on other fitness components. In this paper, we develop an analytical model for the evolution of autosomal drivers that is motivated by the neocentromere drive system found in maize. In particular, we model both the transmission advantage and deleterious fitness effects on seed viability, pollen viability, seed to adult survival mediated by maternal genotype, and seed to adult survival mediated by offspring genotype. We derive general, biologically intuitive conditions for the four most likely evolutionary outcomes and discuss the expected evolution of autosomal drivers given these conditions. Finally, we determine the expected equilibrium allele frequencies predicted by the model given recent estimates of fitness components for all relevant genotypes and show that the predicted equilibrium is within the range observed in maize land races for levels of drive at the low end of what has been observed.

Mendel’s law of equal segregation is almost always true, such that half the gametes contain one allele and half contain the other. The reason is that each meiosis results in two meiotic products that contain one allele and two that contain the other. However, there are a few exceptions to equal segregation, often termed meiotic drivers, in which one allele is overrepresented in gametes. Such meiotic drivers include autosomal killers (*e.g.*, the *t* locus in mice; Lyon 2003), sex-ratio drivers [*e.g.*, killer X chromosomes in *Drosophila*; see Jaenike (2001)], and others (Burt and Trivers 2006; Lindholm *et al.* 2016). The mechanisms causing meiotic drive often do not occur during meiosis *per se*, but instead happen either before (*e.g.*, sex chromosome drive in female wood lemmings; Fredga *et al.* 1976) or, more usually, after meiosis. Drivers that act postmeiotically often involve the destruction of sperm (*e.g.*, most sex chromosome drivers in Diptera; Jaenike 2001).

Drive mechanisms that occur during meiosis itself are likely rare for two reasons. First, a driver that targets a homolog during meiosis must contend with the fact that there are two independent cell divisions with random chromosome alignment, such that a driver might inadvertently target itself [see Haig and Grafen (1991)]. Second, meiosis is often symmetric, such that all meiotic products become gametes, which reduces the chance that mechanisms acting during meiosis will lead to overrepresentation of drivers in the next generation. These two characteristics of meiosis may have evolved to minimize the possibility of drive during meiosis [Leigh 1971, 1977; but see Úbeda and Haig (2005)]. However, there are many species, especially in plants and vertebrates, in which female meiosis is asymmetric, such that only one product of meiosis becomes an egg nucleus and the others become polar bodies that do not contribute genetically to the next generation. This sets the stage for mechanisms that cause chromosomes to preferentially end up in the egg nucleus as opposed to the polar bodies. Such mechanisms act during meiosis and represent meiotic drivers in the strict sense of the term.

In 1942, Marcus Rhoades described the aberrant segregation of a purple (*vs.* yellow) marker that was present on chromosome 10 (Rhoades 1942). A particular copy of chromosome 10, which he termed Abnormal chromosome 10 (Ab10), was transmitted through ovules to ∼75% of the progeny in Ab10/N10 heterozygotes (N10 is the normal chromosome 10). There was no segregation distortion through pollen. Recent data indicate that the mechanism involves at least two components (Lowry 2015; R. K. Dawe, E. G. Lowry, J. I. Gent, M. C. Stitzer, and D. M. Higgins, unpublished results), including a large heterochromatic “knob” region and a closely linked complex of kinesin genes, the *Kinesin driver* (*Kindr*) complex. The heterochromatic region is presumed to act as a binding site for KINDR proteins, causing the poleward motility of the knob and linked *Kindr* loci. The resulting dramatic movement of knobs, dubbed neocentromeres, was one of the first properties observed in Ab10 lines (Rhoades and Vilkomerson 1942). Meiotic drive based on unequal centromere activity has also been observed in one other plant species, *Mimulus gutattus* (Fishman and Willis 2005; Fishman and Saunders 2008), and in mice, where Robertsonian fusions/fissions have stronger centromere activity and are able to drive in heterozygous females (Chmátal *et al.* 2014). These examples tangentially support the centromere drive theory for rapid centromere and centromere-binding protein (CenH3 histone) sequence evolution (Malik and Henikoff 2002).

The knob, the *Kindr* complex, and presumably other modifiers of meiotic drive occur in a nonrecombining haplotype at the end of the long arm of chromosome 10 (the Ab10 haplotype). The Ab10 haplotype is far enough from the centromere that crossing over is effectively guaranteed, maximizing the likelihood of heteromorphic chromosomes with one copy of the Ab10 haplotype and one copy of N10 [this MII segregation pattern should occur two-thirds of the time; Beadle 1946; reproduced in Davis and Perkins (2002)]. When neocentromere activity acts on heteromorphic chromosomes (Figure 1), knob-containing chromatids from an MII segregation pattern will be preferentially transmitted to the outermost positions of the meiotic spindle, one of which will become the egg nucleus. When Ab10 and N10 are in different meiosis I products (an MI segregation pattern), there is a one in two chance that Ab10 will be transmitted to the egg nucleus. Thus, an Ab10/N10 heterozygote is expected to produce at most 2/3 × 1 + 1/3 × 1/2 = 5/6 (= 83%) Ab10 gametes. Ab10 chromosomes exhibit drive that is slightly below this maximum, presumably due to some combination of insufficient crossing over, such that MII segregation patterns occur less often than two-thirds of the time, or failure to exhibit complete drive when there is an MII pattern.

**Figure 1:**
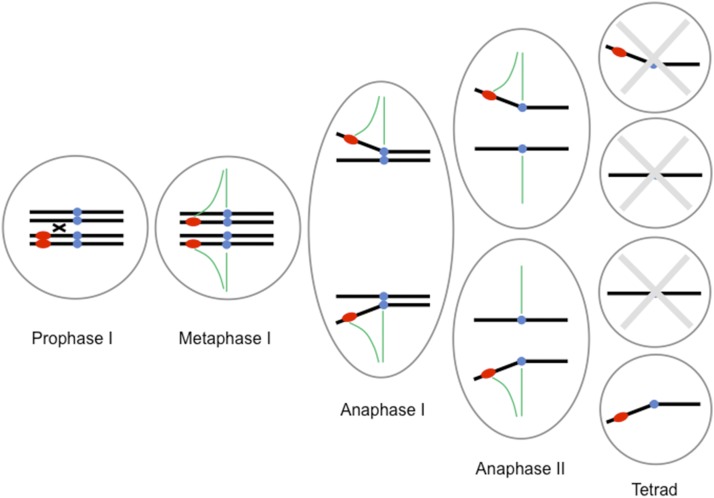
The manner in which drive works in Ab10/N10 heterozygotes. One or more crossovers results in each MI product containing an Ab10 and N10 chromatid (a meiosis II segregation pattern). The Ab10 chromatids are then preferentially transmitted to the outermost positions of the meiotic spindles, and the bottom cell of the tetrad will become the egg nucleus. When there is an MI segregation pattern, Ab10 will end up in the egg 50% of the time, *i.e.*, at random. Chromosomes are colored black, knobs are red, centromeres are blue, and spindle fibers are green. Ab10, abnormal chromosome 10; MI, meiosis I; N10, normal chromosome 10.

The Ab10 haplotype spans several megabases and contains hundreds of genes required for normal growth and development. Multiple inversions within the haplotype severely restrict recombination with N10 (Mroczek *et al.* 2006) such that deleterious alleles are expected to accumulate in this large region. Although Ab10 is a strong driver, showing up in 62–79% of ovules of heterozygotes, it is found at frequencies of only 0–33% in natural populations (Kanizay *et al.* 2013b). Similar low frequencies are found for other extant drive systems (Burt and Trivers 2006). The balance between drive and deleterious fitness consequences has been extensively modeled for autosomal drivers (Hiraizumi *et al.* 1960; Lewontin 1968; Hartl 1970; Fishman and Kelly 2015). These models indicate that autosomal drivers are expected to rapidly sweep to fixation unless they have strong pleiotropic, deleterious fitness costs. A stable polymorphism is most likely when deleterious fitness consequences are recessive. The reason is that at low frequency, the driver is usually heterozygous and thus pays no fitness costs and invades due to drive. However, at high frequency, the driver occurs more frequently in homozygotes where the deleterious fitness effects are expressed, preventing fixation.

A recent companion study (Higgins *et al.* 2017) has examined multiple components of fitness for maize plants heterozygous or homozygous for the Ab10 chromosome. Fitness costs have been found for pollen viability, ovule viability, and seed size. In this study, we model these fitness components in conjunction with meiotic drive, assuming that seed size is a maternal effect that affects survival from seed to flowering. We also consider an additional effect on the survival from seed to flowering. In particular, we assume that it is affected by the genotype of the offspring in addition to the maternal effect through seed size. The goal is to determine the behavior of the general model in terms that maximize biological understanding, and to specifically determine whether the estimated fitness components predict an equilibrium frequency of the Ab10 chromosome that is consistent with what is observed in natural populations of maize.

## Methods

We model the system as a single locus, at which two alleles are segregating, Ab10 and N10. The N10/N10 homozygote is the wild-type genotype and has maximal fitness for all components of fitness. The heterozygote, Ab10/N10, experiences drive during ovule production, such that a proportion (1 + *d*)/2 of the ovules contain the Ab10 allele and (1 − *d*)/2 of the ovules contain the N10 allele (0 ≤ *d* ≤ 1). In addition to affecting segregation in ovules in heterozygotes, the Ab10 allele has four other fitness effects (Table 1). First, Ab10 reduces pollen viability, such that Ab10/Ab10 homozygotes produce (1 − *m*) as much viable pollen, and Ab10/N10 heterozygotes produce (1 − *h_m_ m*) as much viable pollen as N10/N10 homozygotes (0 ≤ *h_m_*, *m* ≤ 1). Second, Ab10 reduces ovule viability, such that Ab10/Ab10 homozygotes produce (1 − *f*) as many viable ovules, and Ab10/N10 heterozygotes produce (1 − *h_f_ f*) as many viable ovules as N10/N10 homozygotes (0 ≤ *h_f_*, *f* ≤ 1). Third, Ab10 reduces seed viability through seed size, such that seeds produced by Ab10/Ab10 homozygotes are (1 − *s*) as viable, and seeds produced by Ab10/N10 heterozygotes are (1 − *h_s_ s*) as viable as seeds produced by N10/N10 homozygotes (0 ≤ *h_s_*, *s* ≤ 1). Note that this is essentially a maternal effect, whereby plants carrying Ab10 invest fewer resources in seeds than N10 individuals. Fourth, Ab10 reduces survival from seed to flowering, such that Ab10/Ab10 homozygotes survive at rate (1 − *v*), and Ab10/N10 heterozygotes survive at rate (1 − *h v*) in comparison to N10/N10 homozygotes (0 ≤ *h*, *v* ≤ 1).

**Table 1:** The drive and relative fitness parameters for each of the three genotypes

Genotype	Ab10 Ovules	N10 Ovules	Ab10 Pollen	N10 Pollen	Pollen Viability	Seed Viability	Survival*^a^* (Seed Size)	Survival*^a^* (Seed Genotype)
Ab10 Ab10	1	0	1	0	1 − *m*	1 − *f*	1 − *s*	1 − *v*
Ab10 N10	(1+d)/2	(1−d)/2	1/2	1/2	1 − *h_m_ m*	1 − *h_f_ f*	1 − *h_s_ s*	1 − *h v*
N10 N10	0	1	0	1	1	1	1	1

All parameters in the table lie in (0, 1). Ab10, abnormal chromosome 10; N10, normal chromosome 10.

a From germination to flowering.

Letting *p_f_* and *p_m_* represent the frequency of the Ab10 allele in one generation and $p_{f}^{\prime }$ and $p_{m}^{\prime }\;$its frequency in the next generation in ovules and pollen, respectively, these assumptions result in the following recursions:

$$p_{f}^{\prime }=\frac{1}{W_{f}}\left(p_{f}p_{m}\left(1-f\right)\left(1-s\right)\left(1-v\right)+\left(\frac{p_{f}q_{m}+p_{m}q_{f}}{2}\right)\left(1-h_{f}f\right)\left(1-h_{s}s\right)\left(1-hv\right)\left(1+d\right)\right),$$ (1a)

$$q_{f}^{\prime }=1-p_{f}^{\prime }$$ (1b)

$$p_{m}^{\prime }=\frac{1}{W_{m}}\left(p_{f}p_{m}\left(1-m\right)\left(1-v\right)+\left(\frac{p_{f}q_{m}+p_{m}q_{f}}{2}\right)\left(1-h_{m}m\right)(1-hv)\right),$$ (1c)

and

$$q_{m}^{\prime }=1-p_{m}^{\prime }$$ (1d)

where

$$W_{f}=p_{f}p_{m}\left(1-f\right)\left(1-v\right)\left(1-s\right)+\left(p_{f}q_{m}+p_{m}q_{f}\right)\left(1-hv\right)\left(1-h_{f}f\right)\left(1-h_{s}s\right)+q_{f}q_{m},$$

$$W_{m}=p_{f}p_{m}\left(1-m\right)\left(1-v\right)+\left(p_{f}q_{m}+p_{m}q_{f}\right)\left(1-h_{m}m\right)\left(1-hv\right)+q_{f}q_{m},$$

$$q_{f}=1-p_{f},\;\mathrm{and}\;q_{m}=1-p_{m}.$$

### Data availability

The authors state that all data necessary for confirming the conclusions presented in the article are represented fully within the article.

## Results

**Figure 2:**
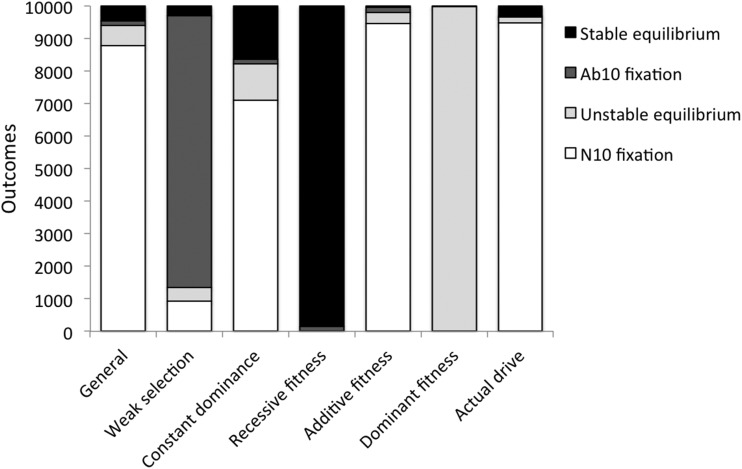
Global behavior of the model for randomly drawn values of parameters for different scenarios. Shown is the number of parameter combinations giving each of the four possible outcomes when global behavior is fully predicted by the sign of the eigenvalues in Equations (2) and (3). These outcomes represent 99.4–100% of parameter sets across the different scenarios. In the general scenario, all parameters can take any value in their range. For weak selection, each fitness component of the Ab10/Ab10 homozygote is within 90% of the N10/N10 homozygote. For constant dominance, the dominance parameters of the Ab10 chromosome across all fitness components are identical, such that *h* = *h_f_* = *h_s_* = *h_m_*. The next three scenarios (recessive, additive, and dominant fitness) assume that the Ab10 chromosome has fitness effects that are either fully recessive (Ab10/N10 heterozygote fitness is the same as N10/N10 homozygote), additive (Ab10/N10 heterozygote fitness is exactly intermediate between the two homozygotes), or fully dominant (Ab10/N10 heterozygote fitness is the same as the Ab10 Ab10 homozygote). In each of these three, all fitness effects again have the same dominance parameter (*h* = *h_f_* = *h_s_* = *h_m_*). The last column (actual drive) restricts the drive parameter to the range observed in maize land races (0.2 ≤ *d* ≤ 0.6). The four possible outcomes are: N10 goes to fixation regardless of the starting frequency (N10 fixation), Ab10 goes to fixation regardless of the starting frequency (Ab10 fixation), N10 or Ab10 goes to fixation depending on the starting frequency (unstable equilibrium), or Ab10 and N10 reach an internal equilibrium frequency regardless of the starting frequency (stable equilibrium). Not shown are the very few parameter combinations in which more than one internal equilibrium exists. These only occur in the general, constant dominance, and additive fitness scenarios for 37, 28, and 69 parameter combinations, respectively. The observed outcomes clearly show that weak selection favors Ab10 fixation and that recessive fitness effects strongly favor an internal equilibrium in which both the N10 and Ab10 chromosomes segregate. In all other cases, there are relatively few parameter combinations that allow invasion of an Ab10 driver. Ab10, abnormal chromosome 10; N10, normal chromosome 10.

To solve for the equilibrium frequencies of the Ab10 allele in ovules and pollen, ${\hat{p}}_{f}$ and ${\hat{p}}_{m},$ we set $p_{f}^{\prime }\;$ = *p_f_* = ${\hat{p}}_{f}$ and $p_{m}^{\prime }\;$ = *p_m_* = ${\hat{p}}_{m}.$ Using Mathematica (version 9; Wolfram Research, Inc. 2012) to determine the number of equilibria for several hundred thousand randomly drawn sets of parameter values indicates that, in addition to the two fixation equilibria, ${\hat{p}}_{f}$ = ${\hat{p}}_{m}$ = 0 and ${\hat{p}}_{f}$ = ${\hat{p}}_{m}$ = 1, up to three internal equilibria (0 ≤ ${\hat{p}}_{f},$ ${\hat{p}}_{m}$ ≤ 1) can exist. However, in the vast majority of cases, zero or one internal equilibrium exists (Figure 2). When there is at most one internal equilibrium, its existence and stability can be inferred from the eigenvalues of the stability matrices associated with each of the fixation equilibria (Otto and Day 2007).

Equations (1) were used to generate the stability matrices for the two fixation equilibria, ${\hat{p}}_{f}$ = ${\hat{p}}_{m}$ = 0 and ${\hat{p}}_{f}$ = ${\hat{p}}_{m}$ = 1, from which the leading eigenvalues were determined (Otto and Day 2007). The leading eigenvalue associated with the introduction of the Ab10 allele into a population fixed for N10, $\lambda_{0},$ is

$$\lambda_{0}=\left(1-hv\right)\left[\frac{\left(1-h_{f}f\right)\left(1-h_{s}s\right)\left(1+d\right)}{2}+\frac{\left(1-h_{m}m\right)}{2}\right],$$ (2)

and the leading eigenvalue associated with the introduction of the N10 allele into a population fixed for Ab10, $\lambda_{1},$ is

$$\lambda_{1}=\frac{\left(1-hv\right)}{\left(1-v\right)}\left[\frac{\left(1-h_{f}f\right)}{\left(1-f\right)}\frac{\left(1-h_{s}s\right)}{\left(1-s\right)}\frac{\left(1-d\right)}{2}+\frac{\left(1-h_{m}m\right)}{2\left(1-m\right)}\right].$$ (3)

The eigenvalues have the same general structure for both fixation equilibria and have simple biological interpretations. The term to the left of the square brackets is the relative seed to flowering survival of the heterozygote Ab10/N10, (1 − *h v*), relative to the resident homozygote, which is 1 for N10/N10 or (1 − *v*) for Ab10/Ab10 homozygotes. The two terms inside the square brackets specify the relative fitness of the introduced allele through gametes. The first is the relative fitness through ovules (female function) and the second is relative fitness through pollen (male function). The male function term is the relative pollen viability of heterozygotes, $\;\left(1-h_{m}m\right),$ divided by the relative pollen viability of the resident homozygote, which is 1 for N10/N10 homozygotes and (1−m) for Ab10/Ab10 homozygotes, multiplied by transmission of the rare allele through pollen. Transmission of the rare allele through pollen is equal to one-half, regardless of the allele’s identity, since it is found predominantly in heterozygotes and there is no drive in pollen, though such an effect can be simply added (see *Discussion*). The female function term has three parts. First is the relative number of ovules in heterozygotes, $\;\left(1-h_{f}f\right),$ divided by the relative number of ovules of the resident homozygote, which is 1 for N10/N10 homozygotes and (1−f) for Ab10/Ab10 homozygotes. Second is the relative survival of seeds from heterozygotes, $\;\left(1-h_{s}s\right),$ divided by the relative survival of seeds of the resident homozygote, which is 1 for N10/N10 homozygotes and (1−f) for Ab10/Ab10 homozygotes. Third is the transmission of the rare allele through ovules, which includes the drive parameter, and is equal to (1 + *d*)/2, when the Ab10 driver chromosome is introduced into a population fixed for N10 and (1 − *d*)/2 when the N10 chromosome is introduced into a population fixed for the Ab10 driver.

Invasion of the introduced allele requires that the leading eigenvalue is > 1 (Otto and Day 2007). This occurs when the product of the rare allele’s relative viability from seed to flowering, multiplied by its relative fitness through pollen and ovules is > 1, implying that it is contributing more to the next generation in heterozygotes than the resident allele in a homozygote. Since each eigenvalue could be greater or less than 1, the stability analyses predict four possible outcomes of the model:

Outcome 1: $\lambda_{0}$ < 1, $\lambda_{1}$ > 1. N10 will invade when rare. The Ab10 driver cannot invade when rare. For most parameter values, the global behavior is that N10 will invade when rare and then go to fixation. This case occurs when the level of drive, *d*, is small relative to the costs of the driver (*h v*, *h_f_ f*, *h_s_s*, and *h_m_m*).
Outcome 2: $\lambda_{0}$ > 1, $\lambda_{1}$ < 1. The Ab10 driver will invade when rare. N10 cannot invade when rare. For most parameter values, the global behavior is that Ab10 will invade when rare and then go to fixation. This case occurs when the level of drive, *d*, is large relative to the heterozygous and homozygous costs of the driver (*h v*, *h_f_f*, *h_s_s*, *h_m_m*, *v*, *m*, and *f*), *i.e.*, selection on fitness components is weak.
Outcome 3: $\lambda_{0},$ $\lambda_{1}$ < 1. Both equilibria are stable to invasion, *i.e.*, neither allele can invade when rare. For most parameter values, this case corresponds to a single unstable internal equilibrium. Ab10 cannot invade when rare but, if it is sufficiently common, it will go to fixation. Similarly, N10 cannot invade when rare but, if common, it will go to fixation. This case occurs when heterozygous costs (*h v*, *h_f_ f*, *h_s_s*, and *h_m_ m*) are large relative to the level of drive, *d*, but the costs in homozygotes (*v*, *f*, and *m*) are not much larger than in heterozygotes. Dominant fitness effects (*h*, *h_f_*, and *h_m_* close to 1) make this outcome more likely.
Outcome 4: $\lambda_{0},$ $\lambda_{1}\;$> 1. Both equilibria are unstable to invasion, *i.e.*, either allele can invade when rare. For most parameter values, this case corresponds to a single stable internal equilibrium. This case occurs when heterozygous costs (*h v*, *h_f_ f*, *h_s_ s*, and *h_m_ m*) are small relative to the level of drive, *d*, but the costs in homozygotes (*v*, *f*, and *m*) are large. Recessive fitness effects (*h*, *h_f_*, and *h_m_* close to zero) make this outcome more likely.

To gain insight into the relative likelihood of the above four outcomes, the leading eigenvalues were calculated for randomly drawn parameter values in the general case (0 ≤ *h*, *h_f_*, *h_s_*, *h_m_*, *v*, *f*, *s*, and *m* ≤ 1) and for situations in which selection was assumed to be relatively weak (0 ≤ *v*, *f*, s, and *m* ≤ 0.1), or the Ab10 driver was assumed to be fully recessive (*h* = *h_f_* = *h_s_* = *h_m_* = 0), additive (*h* = *h_f_* = *h_s_* = *h_m_* = 1/2), or fully dominant (*h* = *h_f_* = *h_s_* = *h_m_* = 1) in all of its fitness effects. In addition, we considered the situation in which the drive parameter is constrained to be between 0.2 and 0.6, equivalent to a frequency of the driver allele between 0.6 and 0.8 in gametes of heterozygotes, which is the observed range of drive in Ab10 (Higgins *et al.* 2017). Equations (1) were also used to determine the number of internal equilibria for every combination of randomly chosen parameter values using the numerical solving capabilities of Mathematica (version 9; Wolfram Research, Inc. 2012). Results are shown in Figure 2. In the vast majority of cases, zero or one internal equilibrium exists and so the eigenvalues given in Equations (2) and (3) predict the global behavior of the model, as described above. However, for a very few parameter combinations, two or three internal equilibria can exist. This occurred for < 0.4% of the data sets in the general case, and for < 0.7% of the data sets in the additive fitness case, when it was most prevalent.

**Figure 3:**
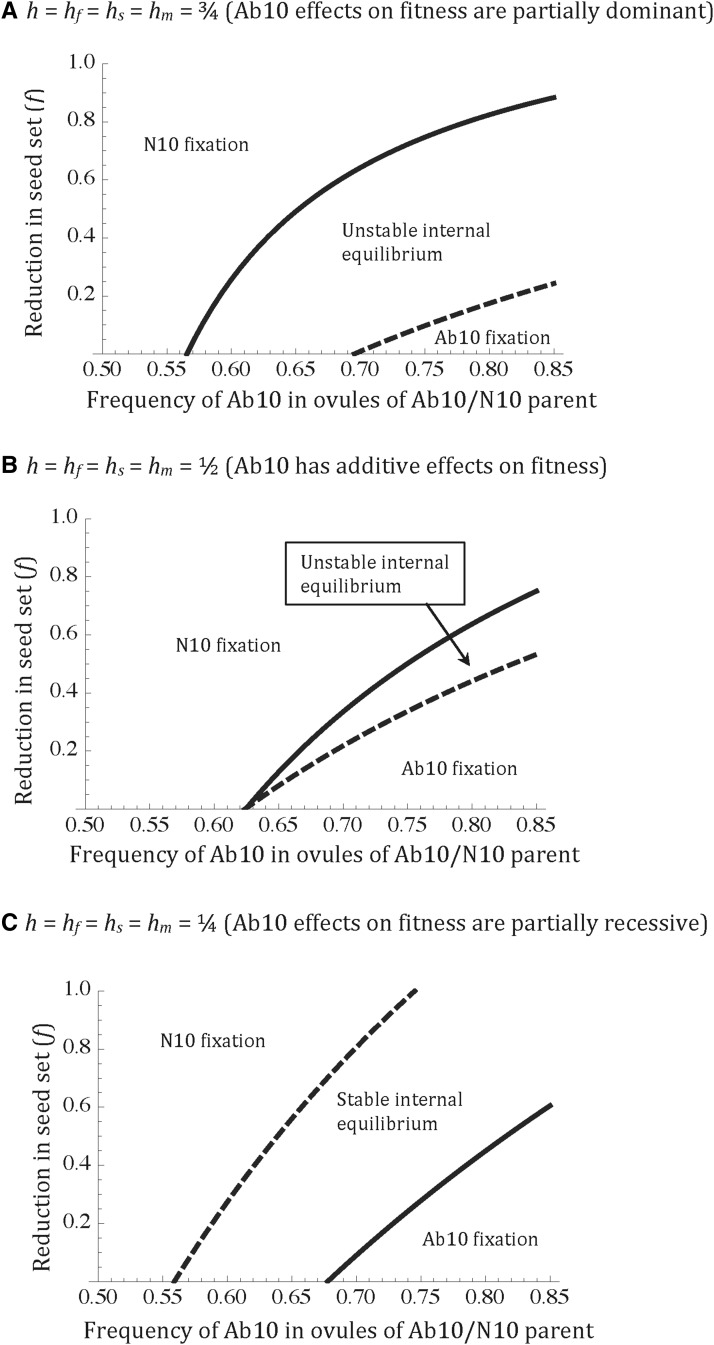
The effect of the degree of dominance on behavior of the model. Lines represent values of drive, expressed as the frequency of Ab10 in ovules of heterozygotes, which is equal to one-half (1 + *d*), and seed set reduction in homozygotes, *f*, at which the eigenvalues associated with fixations are equal to one. The solid line is for $\lambda_{1}$: above this line $\lambda_{1}$ is > 1 (Ab10 fixation can be invaded by N10) and below it is < 1 (Ab10 fixation cannot be invaded by N10). The dashed line is for $\lambda_{0}$: above the line $\lambda_{0}$ is < 1 (N10 fixation cannot be invaded by Ab10) and below the line it is > 1 (N10 fixation can be invaded by Ab10). In most cases, these eigenvalues predict global behavior (see Figure 2). The expected global behavior is given for each of the delineated regions. The three panels are for different levels of dominance of fitness components, including partial dominance (A), additivity (B), or partial recessivity (C). The more dominant the fitness effects of the driver, the less likely that it will invade a population fixed for N10 (*i.e.*, the smaller the region under the dashed line). Drivers with more recessive effects on fitness, on the other hand, and more likely to invade, and more likely to give a stable equilibrium [compare (C) to (A and B)]. In all three panels: *h* = *h_f_* = *h_s_* = *h_m_* (dominance of fitness effects is the same for all fitness components), *m* = 0.05 (5% reduction in pollen viability in Ab10/Ab10 homozygotes), *s* = 0 (no reduction in seed viability due to maternal genotype), and *v* = 0.2 (10% reduction in viability from germination to adult in Ab10/Ab10 homozygotes). Ab10, abnormal chromosome 10; N10, normal chromosome 10.

There are several patterns that can be seen in Figure 2. First, in the general case, an Ab10 driver only invades a population fixed for N10 for ∼6% of randomly drawn parameter values (Outcomes 2 and 4). This makes sense when one considers that drivers can cause deleterious effects on three components of fitness (seed viability, pollen viability, and survival from seed to flowering), with survival from seed to flowering affected by both indirect maternal effects (seed reserves are deposited by the mother) and direct effects on growth after seed reserves are exhausted. If any of the reductions is large for any of the fitness effects in the heterozygote, then the driver will be prevented from entering the population. Second, if selection is weak, such that fitness effects are each quite small, < 10%, then a driver will invade a population (and fix) for ∼86.5% (and 83.5%) of parameter values. Third, invasion of a driver is very likely when its fitness effects are recessive. When the effects of the driver are fully recessive for all three fitness components (*h* = *h_f_* = *h_m_* = 0), it is always able to invade a population fixed for N10 because it does not express fitness costs in the heterozygote, though it only goes to fixation for ∼1.5% of parameter values. Thus, recessive fitness costs of the Ab10 driver favor a stable internal equilibrium (∼98.5% of outcomes). Fourth, when the fitness effects of the driver are fully dominant, few parameter values allow invasion of a population fixed for N10 (0.14% of outcomes). Interestingly, when the driver is fully dominant, then the N10 allele has no fitness advantage when invading a population fixed for Ab10. Thus, if a driver with dominant fitness effects can get to high frequency through a founder event or something similar, then it will sweep to fixation. Together, these patterns suggest that autosomal meiotic drivers that are polymorphic in a population, such as the Ab10 chromosome in maize, are likely to be recessive across multiple fitness components and have large fitness effects in homozygotes, as previously found (Hartl 1970; Fishman and Kelly 2015). A graphical illustration of the effect of dominance on the evolutionary outcome for an autosomal driver is shown in Figure 3. For high levels of dominance, an internal equilibrium, if it exists, is likely to be unstable and thus long-term persistence of a driver is unlikely. For drivers that are partially recessive in their fitness effects, an internal equilibrium, if it exists, is more likely to be stable such that long-term persistence of the driver is a more common outcome.

### Ab10 frequency in maize land races

Ab10 varies in frequency from 0 to 33% across maize land races (Kanizay *et al.* 2013b). We wished to determine whether fitness values associated with the three genotypes (Ab10/Ab10, Ab10/N10, and N10/N10) are sufficient to explain the observed range of population frequencies. In a field experiment containing all three genotypes, seed production, seed weight, and pollen viability were measured, as was the degree of drive of nine different Ab10 strains (Higgins *et al.* 2017). Ab10 had a modest effect on pollen viability, causing a 1 and 6% reduction in Ab10/N10 heterozygotes and Ab10/Ab10 homozygotes, respectively, though the heterozygous effect was not significantly different from the N10/N10 homozygote. However, Ab10 had a large effect on seeds. Seed production was reduced by 13 and 65%, and seed mass was reduced by 6 and 19% in Ab10/N10 heterozygotes and Ab10/Ab10 homozygotes, respectively. Drive varied from 62 to 79% across nine Ab10 chromosomes. However, it was not uniformly distributed. Seven Ab10 chromosome are transmitted to ∼76% of ovules (range 73–79%), whereas the other two are transmitted to ∼62% of ovules.

**Figure 4:**
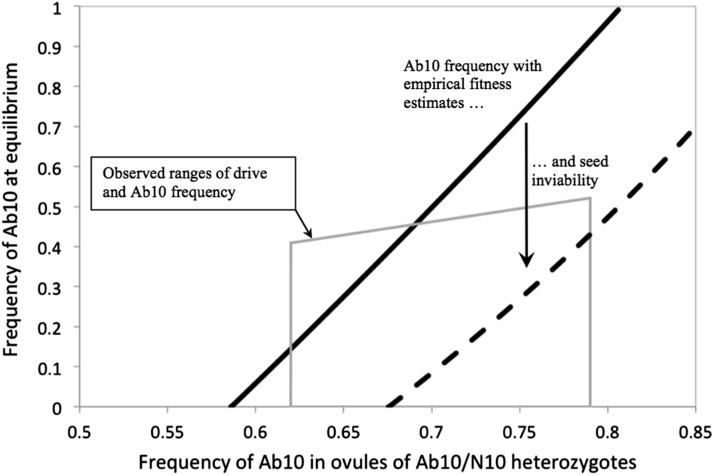
Predicted frequency of Ab10 in ovules at the stable equilibrium using fitness parameters from a field experiment. The solid line is equilibrium for the average values of seed set, seed size (seed viability), and pollen viability measured in Higgins *et al.* (2017) (*f* = 0.650, *s* = 0.190, *m* = 0.063, *h_f_* = 0.212, *h_s_* = 0.327, and *h_m_* = 0.176). The dashed line is the equilibrium after including hypothetical reductions in seed viability due to offspring genotype (*v* = 0.3 and *h* = 0.238) to illustrate how the predicted equilibrium changes. The gray box delineates the minimum and maximum values of drive and Ab10 frequencies observed in maize land races. Note that the maximum observed Ab10 frequency increases with the level of drive because frequencies were observed in adult plants, and the model censuses ovules (and pollen), which is postdrive. Ab10, abnormal chromosome 10; N10, normal chromosome 10.

Using these fitness component estimates for the three genotypes, we determined the frequency of Ab10 at equilibrium for levels of drive, *d*, that varied between 0 and two-thirds, corresponding to a frequency of Ab10 in ovules of heterozygous mothers of 0.5 to 0.83, which is the full range of drive given its mechanism. To determine Ab10 frequency at equilibrium, we iterated Equation (1) until an equilibrium was reached. We also checked for global behavior by determining the number of equilibria using Mathematica (version 9; Wolfram Research, Inc. 2012). We assumed that seed mass affects plant viability such that a reduction in seed mass relative to N10/N10 results in an identical relative reduction in plant survival to reproduction. We note that an effect on plant fecundity caused by plant size due to seed size is mathematically equivalent to a maternal effect on seed to adult viability. The assumption that reduced seed size reduces fitness has mixed support in maize. Some studies have found that reduced seed size decreases seedling emergence from soil under stressful conditions (Graven and Carter 1990), reduces adult plant size (Hunter and Kannenberg 1972), or reduces germination rate after storage (Moreno-Martinez *et al.* 1998). However, it has also been shown that small seed size is associated with increased germination rate under osmotic stress (Muchena and Grogan 1977), or has no effect on germination rate, plant size, or yield under less stressful conditions (Graven and Carter 1990). Since the direct effects of Ab10 on survival to maturity were not measured, we assumed that this fitness component was absent, *i.e.*, *v* = 0. Our findings are shown in Figure 4: the solid black curve is the expected frequency in ovules under different drive parameters, and the solid gray lines are the observed ranges of Ab10 frequency and level of drive. Note that since Ab10 population frequency was measured in adult plants and the model censuses Ab10 frequency in newly formed ovules, adult frequency had to be converted into ovule frequency, which depends on the level of drive. The maximum observed frequency (33%) thus corresponds to frequencies in ovules that are larger for higher levels of drive (Figure 4). For low levels of drive, *d*, below ∼0.173, corresponding to Ab10 frequencies of 0.586 in ovules of Ab10/N10 heterozygotes, the N10 fixation is globally stable. For levels of drive between 0.173 and 0.619, there is a globally stable internal equilibrium. For levels of drive above 0.619, corresponding to Ab10 frequencies of 0.810 in ovules of Ab10/N10 heterozygotes, which is near the maximum possible value of 0.83, Ab10 is expected to fix. Figure 4 illustrates that the stable equilibrium frequency predicted by the model falls in the observed range (0–33%) when Ab10 frequencies in ovules of Ab10/N10 heterozygotes fall between 0.586 and 0.691 (0.173 ≤ *d* ≤ 0.382). These values are indistinguishable from the value of meiotic drive observed for two of the nine Ab10 accessions (62%), but below the values seen for the other seven Ab10 chromosomes extracted from different land races (73–79%).

The fact that a stable equilibrium usually exists when the fitness values are equal to those measured in the field experiment is reassuring, given how seldom randomly drawn parameters exhibit a stable internal equilibrium (Figure 2). It is also reassuring that the observed frequency of the driver in land races corresponds to a level of drive that is within the observed values. However, it is disappointing that the level of drive must fall toward the low end of the observed range and that the observed allele frequencies require a relatively narrow range of drive, *i.e.*, the equilibrium frequency changes rapidly as the level of drive changes. In the experimental work, seed to adult survival was not measured and so the direct effect of Ab10 genotype on this component of fitness was omitted from Figure 4. However, if we add this fitness component, assuming that the strength of selection and dominance are the average of the other measured fitness components, we find that the expected frequency would fall well within the observed range (Figure 4 dashed line).

## Discussion

Like other selfish genetic elements that segregate in natural populations, Ab10 has deleterious effects on fitness. These effects are large in homozygotes and partially recessive so that heterozygotes also show reductions in fitness. Our model indicates that these fitness costs are sufficient to lead to the maintenance of the driver in populations at frequencies that are similar to those observed in land races. The reason that Ab10 is not lost in the face of these large fitness effects is because it is such an efficient driver. Weaker drivers that cause distortion of only a few percent would not be able to invade, or, if already at high frequency, would be rapidly lost if they expressed similar fitness costs (Figure 4). Our model also indicates that the observed equilibrium frequency of Ab10 in land races is lower than predicted given our fitness estimates. There are at least two nonexclusive explanations. First, the effect of Ab10 genotypes on the fitness components were measured for a single Ab10 driver in a single genetic background under controlled field conditions in Iowa (Higgins *et al.* 2017). Measuring fitness effects of different Ab10 drivers in different genetic backgrounds, or geographic locations or environmental conditions, would likely not give the same parameter estimates. Second, there may be at least one additional, unmeasured fitness cost associated with Ab10 that could explain its current frequency in populations. One possibility is a direct effect of Ab10 genotype on seed to adult viability. Addition of this fitness effect, assuming that Ab10/N10 heterozygotes have a 7% reduction and Ab10/Ab10 homozygotes have a 24% reduction in viability, which are the averages for other fitness components, is sufficient to cause equilibrium frequencies to be indistinguishable from those observed in nature (Figure 4, dashed line).

Besides a direct effect on seed viability, there are other possible fitness components that might be affected by the Ab10 chromosome. In previous studies, it was assumed that the Ab10 chromosome might harbor deleterious mutations that resulted in a direct reduction in pollen viability, such that Ab10 haploid pollen grains were less viable that N10 pollen grains [Rhoades 1942; but see Rhoades and Dempsey (1985)]. This would result in segregation distortion in Ab10/N10 heterozygotes such that Ab10 pollen would be underrepresented relative to N10 pollen. However, recent segregation analysis through pollen in Ab10/N10 plants demonstrates that there is no haploid-expressed reduction in pollen viability (Higgins *et al.* 2017), which is why we did not include this effect in the model. We note that adding this fitness component to our model would be straightforward. A haploid pollen viability effect term would simply replace the 0.5 in the male function term in the stability conditions, making it straightforward to predict the global behavior of the model.

The fitness component estimates we used to determine equilibrium frequency were for a single Ab10 chromosome compared to a single N10 chromosome type in a single maize genetic background, in a single location for most measures. Maize is a highly diverse species (Nannas and Dawe 2015) and so it is likely that Ab10 will perform differently on different genetic backgrounds, or in different environments. We know that the level of meiotic drive exhibited by Ab10 can be affected by the N10 chromosome that it is driving against (Kanizay *et al.* 2013a). One variant of chromosome 10 called K10L2 carries a knob at the end that competes with the neocentromere activity of Ab10 and reduces meiotic drive by nearly half (Kanizay *et al.* 2013a). In addition, unlike many other drivers, Ab10 has substantial SNP variation across isolates (Higgins *et al.* 2017). This variation affects the strength of drive, sometimes substantially, so that two Ab10 chromosomes are weaker drivers, found in only 62% of ovules of Ab10/N10 heterozygotes, which is considerably lower than the ∼75% seen for seven other Ab10 chromosomes. There is also pronounced cytological and genetic variation among haplotypes (Kanizay *et al.* 2013b), further suggesting that different Ab10 chromosomes may also differ in their fitness costs. Different fitness values will change the predicted Ab10 equilibrium frequency. A next step is to measure fitness effects for different Ab10s in their normal genetic backgrounds, preferably in the geographic locations where they occur.

The *M. guttatus* centromere-associated driver also drives in females during meiosis (Fishman and Willis 2005; Fishman and Saunders 2008; Fishman and Kelly 2015). This driver exhibits extremely high levels of drive in interspecific crosses (with *M. nasutus*), being found in over 90% of progeny of an F1 hybrid through ovules, and less extreme drive within *M. guttatus*, found in ∼58% of ovules of a heterozygote. It is not yet understood how this driver achieves meiotic drive, though the mechanism is different from Ab10, since the observed level of drive in the interspecific cross exceeds the maximum possible level of 83% for the Ab10 mechanism (see *Introduction*). The *Mimulus* driver also has deleterious fitness effects: a recessive effect on pollen viability of 19% (*i.e.*, *m* = 0.19 and *h_m_* = 0), and a recessive effect on seed number of 21% (*i.e.*, *f* = 0.21, *h_f_* = 0). Indirect evidence suggests that there are no effects of the driver on seed viability (Fishman and Kelly 2015). With these values, the observed frequency of the driver (0.3–0.42) in the natural population in which it occurs was found to be similar to the predicted frequency at equilibrium (0.25–0.40) (Fishman and Kelly 2015).

What is the likely cause of the deleterious fitness effects? The Ab10 and *Mimulus* neocentromere drivers, like other drive systems (Burt and Trivers 2006), contain large inversions, which effectively reduce recombination rates in the drive region. In the absence of recombination, Muller’s ratchet, genetic hitchhiking, and background selection can all lead to the accumulation of deleterious alleles on the nonrecombining region of the chromosome (Charlesworth and Charlesworth 1998). These deleterious mutations may be contributing to the fitness reduction in genotypes carrying the Ab10 chromosome. If so, some of them must have been present on the drive haplotype since it arose, presumably due to hitchhiking with the driver. Without deleterious effects, especially in homozygotes, Ab10 would have quickly swept to fixation (Figure 2). Additional deleterious mutations that accumulate after the driver has entered a population will contribute to the associated deleterious fitness effects and reduce the equilibrium frequency. Another possibility is that the driver itself causes the deleterious fitness effects. Since the mechanism of drive in Ab10 involves kinesin motors pulling knobs along microtubules during meiosis, there may be negative effects on the efficiency or accuracy of mitosis that are particularly pronounced in homozygotes. This hypothesis remains to be tested.

Our model is substantially similar to the autosomal drive model analyzed by Hartl (1970) and further developed by Fishman and Kelly (2015). Hartl assumed that drive could occur in both females and males (to different degrees), but that the fitness effects of the driver did not differ between males and females. Fishman and Kelly focused on female drive and allowed fitness effects to differ for male and female function in hermaphrodites. They also included inbreeding and found that it substantially reduced the equilibrium frequency of drivers because of the reduction in heterozygote frequency, which is where the driver has its only advantage. Our model includes both fitness effects that are specific to male and female function, such that pollen and ovule viability differ, and fitness effects that affect the whole plant. Importantly, the inclusion of additional fitness effects reduces the likelihood that a driver will invade.

Based on the analysis of our model, we can address how neocentromere drivers, or other autosomal drivers, are expected to evolve once they arise by mutation. Inspection of Figure 2 reveals that, in general, a driving chromosome will sweep to fixation only if its effects on other fitness components are small. Assuming that such drivers can arise reasonably frequently through mutation, genomes should be littered with autosomal drivers of small fitness effects that rapidly swept to fixation and are no longer observable. If, on the other hand, fitness effects are large, a driver will usually be unable to invade. The exception is when fitness effects are partially or fully recessive. In this case, a stable equilibrium is a likely outcome, and it is such drivers that we expect to observe in natural populations. There is thus a severe ascertainment bias in our estimates of the fitness effects of autosomal drivers, since the only ones that are polymorphic within populations are those whose fitness effects are strong and at least partially recessive; all others are not detectable, having either failed to invade or swept to fixation.
